# Supplementary material for: Cloning and Organelle Expression of Bamboo Mitochondrial Complex I Subunits Nad1, Nad2, Nad4, and Nad5 in the Yeast Saccharomyces cerevisiae
Source: Int J Mol Sci. 2022 Apr 6;23(7):4054. doi: 10.3390/ijms23074054 (PMC8999482; doi:10.3390/ijms23074054)
Supplement: Supplementary file 1 [file ijms-23-04054-s001.zip › ijms-1608149-supplementary.pdf]

Supplementary Materials

Tsai et al. 2022

Table S1. The primers used for PCR of mitochondrial NADH:ubiquinone oxidoreductase subunits *nad1*, *nad2*, *nad4*, and *nad5*

	Forward primer (5' to 3')	Reverse primer (5' to 3')
nad1a	ACGTACATAGCTGTTCCAGC	ATATTC$^{T}/_{C}$ACATTATAGCC$^{T}/_{A}$GCAACT
nad1b	GGAGCATTACGATCTGCAGCTCAA	ATTCAGCTTCCGCTTCTGGGAG
nad1c	ATCTCCCAGAAGCGGAAGC	ACGGGGA$^{C}/_{G}$TA$^{C}/_{G}$CCGAGCTA
nad1d	ATGGTCCGGTATTCCCTTGT	TAAGGGAGCCATCGAAAGG
nad1-F,R	ACGTACATAGCTGTTCCAGCG	TTAAGGGAGCCATCGAAAGG
nad1F-*Bam*HI(BH	ATATggatccACGTACATAGCTGTTCCAGCGGAAAT	—
nad1R-*Eco*RIII(E3	—	GAGAgaattcTTAAGGGAGCCATCGAAAGGTGACTA
nad2-F,R	ACTGAAGCTTATGTTCAATCTTTTTTTAGC	CTAGAAGCTTCCAGATATGAACTGAGTGCC
nad2-*Bam*HI-F,R	ATGGATCCAGATGTTCAATCTTTTTTTAGCGG	ATATATGGATCCGAGAAGCTTCCAGATGAAC
nad4a	ATGTTAGAACATTTCTGTGAATGC	AGCCGTAGGTGCCTCTACAT
nad4b	GCCAAATCCTTCTATGGATTGC	TGGTAGAGAAATTCGGCATGGT
nad4c	GTAGCCCATATGAATTTGGTGAC	TTTGCCATGTTGCACTAAGTTAC
nad4-F,R	ATGTTAGAACATTTCAGTGAATGCTATTTCGATC	TCAATGAAATTTGCCATGTTGCACTAAGTTA
nad5a	AGAACGATCTAAAGAGGGTCATAG	TTGGCCAAGTATCCTACAAAG
nad5b	TGCCTTTGCTCGGTAGTT	ATGGTGGAAAAGTCTACTGTTTG
nad5c	AGCTATAAAAGCTATGCTTGTCAAT	CCGAATAGTTAGAGATGCCG
nad5d	TTTGACCTATGCCATGATGCT	GAATATCCGAAACGCAGGAA
nad5e	CCTTTAATACTTTTGGCTCT	TTACTCTTGACTTGACTTATTAAT
nad5-F,R	ATGTATCTACTTATTGTCTTTTTGCCTTT	TTATTCTTGACTTGACTTATTAATAATAAAACTAC
nad5-*Eco*RI-F	ACTGGAATTCATGTATCTACTTATTGTCTTTTTGC	GCTCACCATCCCGGGATATTCTTGACTTGACTTAT
nad5-*Xma*I-R	—	GCTCACCATCCCGGGATATTCTTGACTT

		GACTTAT
pYES2-F448	AACCCCGGATCGGACTACTA	—
pYES2-R857	—	CGCAAATTAAAGCCTTCGAG
pEGFP-F10	CTAGCGCTACCGGACTCAGAT	—
pEGFP-R909	—	TCAGGTTCAGGGGGAGGT
mt-egfp-F,R	AAGCTTATGTTACGTTC	GAACTTCAGGGTCAGCTTGC
M13-F,R	GTTTTCCCAGTCACGAC	TCACACAGGAAACAGCTATGAC
A105T-F,R	CATCACAAGCTTTATGTGGGGATCCAG ATG	CATCTGGATCCCCACATAAAGCTTGTG ATG
pT-nad1F-BH	ATATGGATCCGATGTACATAGCTGTTCCAGC	—
pT-nad1R-E1	—	TATAGAATTCGGCCAAGGGAGCCATCGAA
pY-nad1F-H3	ATATAAGCTTATGTACATAGCTGTTCCAGC	—
pY-nad1R-E1	—	ATATGAATTCCGG AAGGGAGCCATCGAA
nad1-top-A695C	CTCTTTTTTTTTTGGGAGcGTATGCCAA TATGATC	—
nad1-bottom-A695	—	GATCATATTGGCATACgCTCCCAAAAAAA AAAGAG
nad4F,R-EZ	*CACAAGCTTGGTACC*ATGTTAGAACATTT CTGTG	*CGGGCCCGCGGTACC*CAATGAAATTTGC CATG
*Bam*HI-nad2-F,R	ATGGATCCAGATGTTCAATCTTTTTTTA GCGG	ATATATGGATCCGAGAAGCTTCCAGAT GAAC
*Eco*RI-nad5-F	ACTGGAATTCATGTATCTACTTATTGTCT TTTTGC	—
*Xma*I-nad5-R	—	GCTCACCATCCCGGGATATTCTTGACTT GACTTAT

The underlined sequences indicated the restriction enzyme cutting site. F: forward primer, R: reverse primer. BH: *Bam* HI, E1: *Eco* RI, and H3: *Hind* III. The sequences in lower case indicated the mutate nucleotides

Table S2. SC-U media

(A)

SC-U medium	
Chemical	Final concentration
yeast nitrogen base without amino acids	0.67% (w/v)
dropout mix without uracil	0.2 % (w/v)
glucose	2% (w/v)

(B)

SC-U medium for induction	
Chemical	Final concentration
yeast nitrogen base without amino acids	0.67% (w/v)
dropout mix without uracil	0.2 % (w/v)
galactose	2% (w/v)

Table S3. Comparison of succinate oxidation in isolated yeast mitochondria from the yeast transformants.

Yeast transformants	-ADP (state 2)	+ADP (sate 3)	RC ratio sate 3/state 2
	nmole O_2 min^{-1} mg^{-1} protein		
Y	138.88 ± 18.35	342.46 ± 0	2.47
MG	85.42 ± 8.80	184.13 ± 25.20	2.16
MN1G	51.03 ± 11.59	174.91 ± 11.21	3.43
MN4G	59.76 ± 11.21	163.04 ± 41.79	2.73

Note: Data were the mean ± SD of four measurements. Y: yeast-pYES2 transformant; MG: pYES2-mt-egfp transformant; MN1G: yeast-pYES2-mt-nad1-egfp transformant; MN4G: yeast-pYES2-mt-nad4-egfp transformant.

Figure S1A

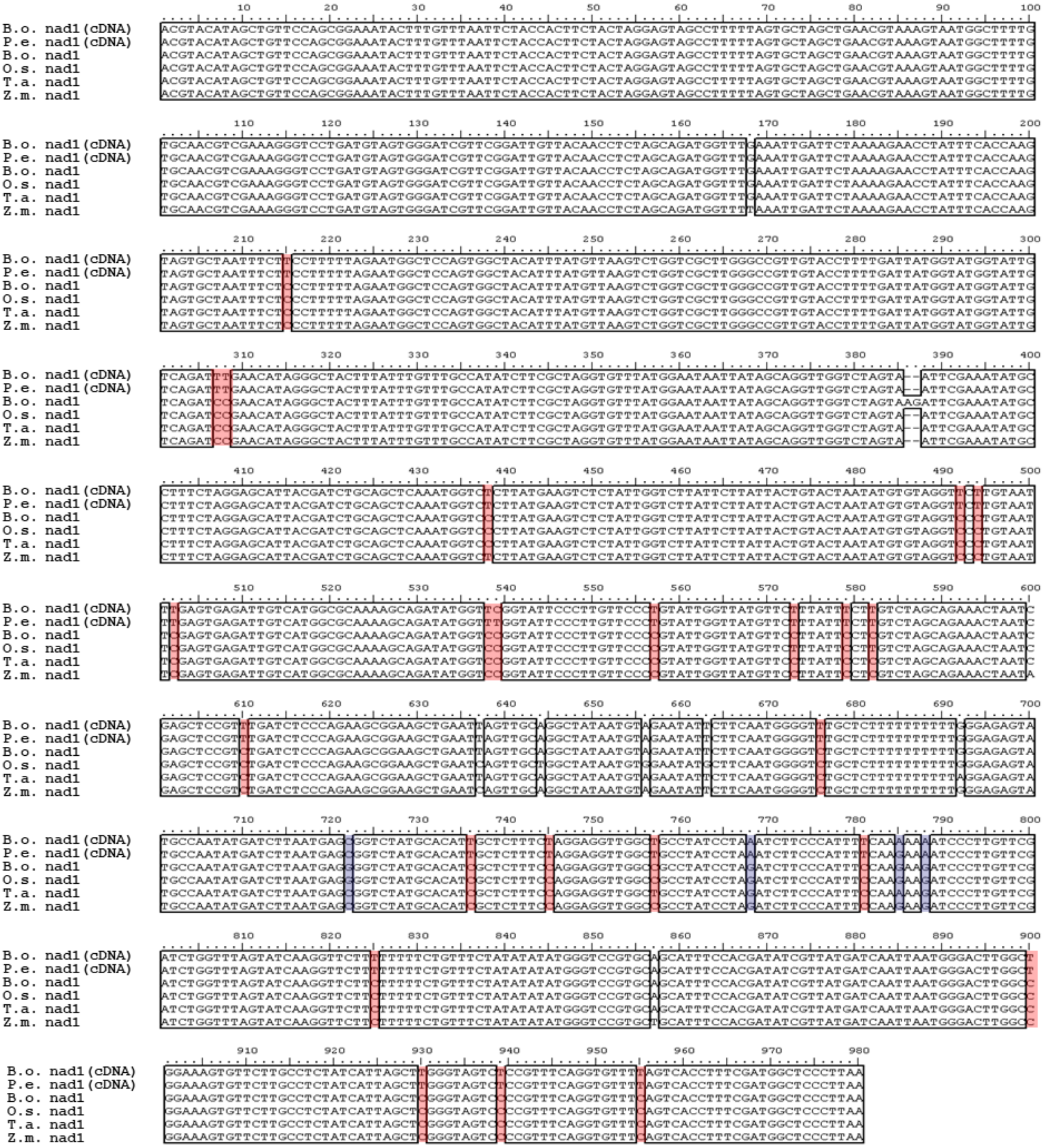

Figure S1B

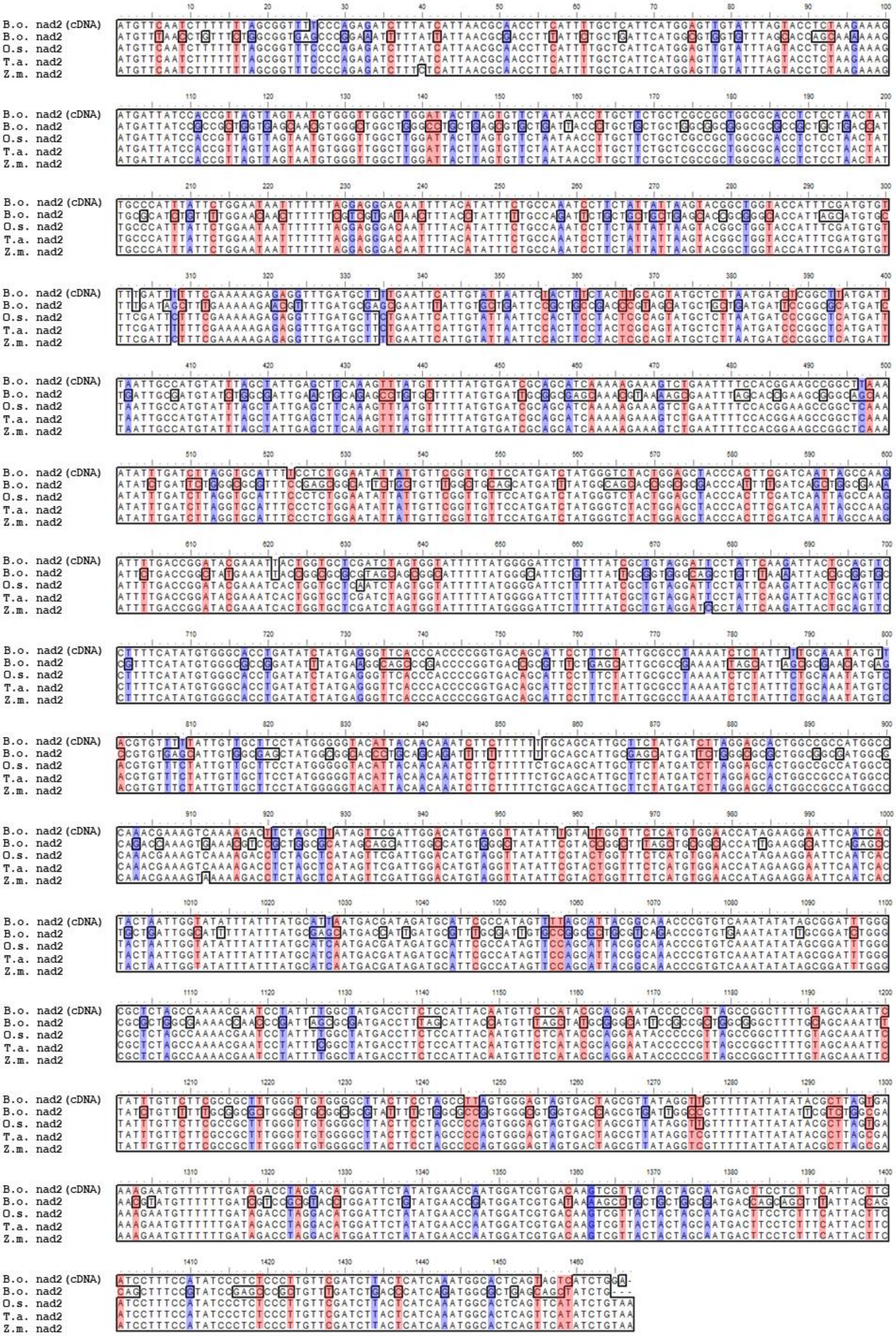

Figure S1C

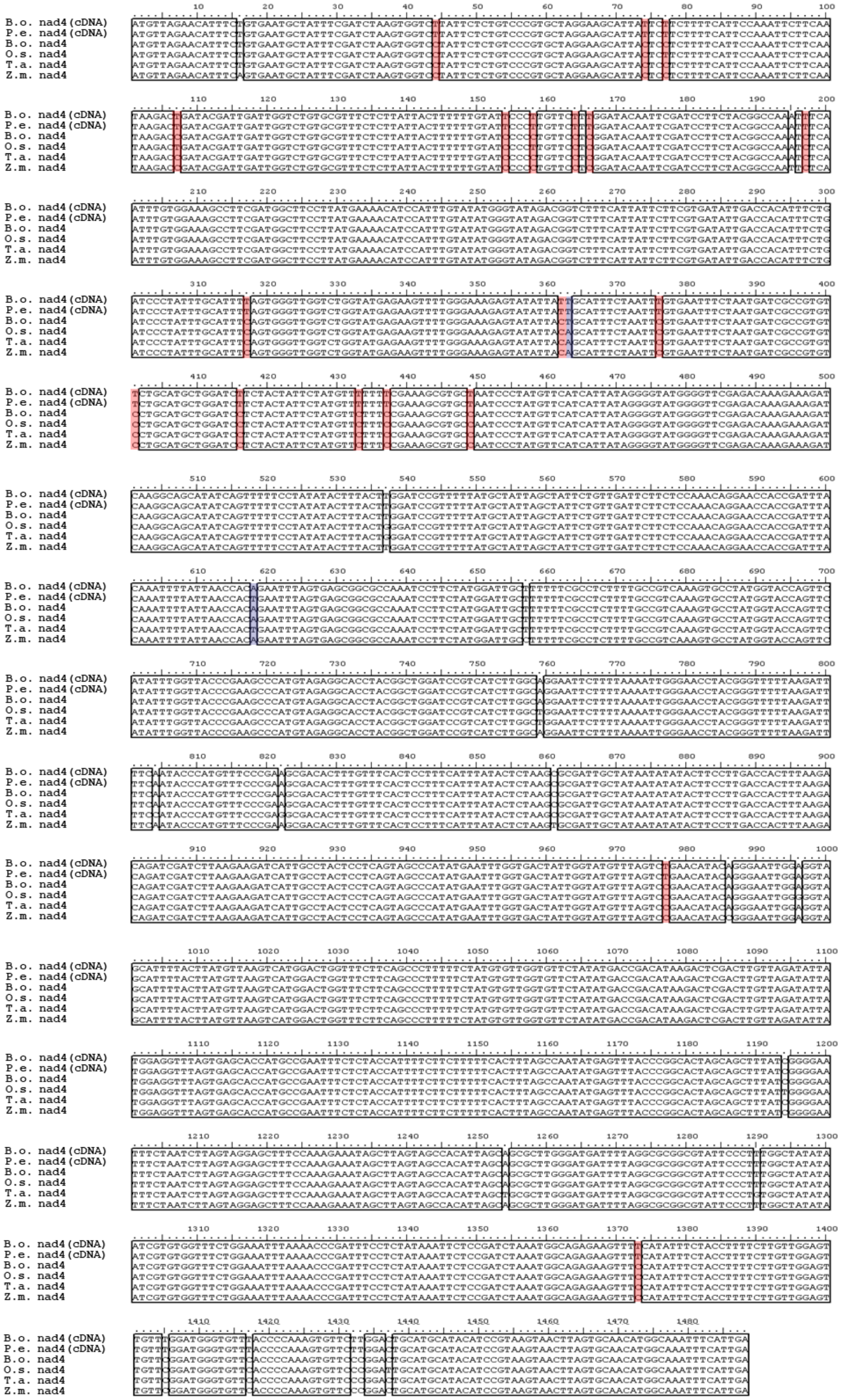

Figure S1D

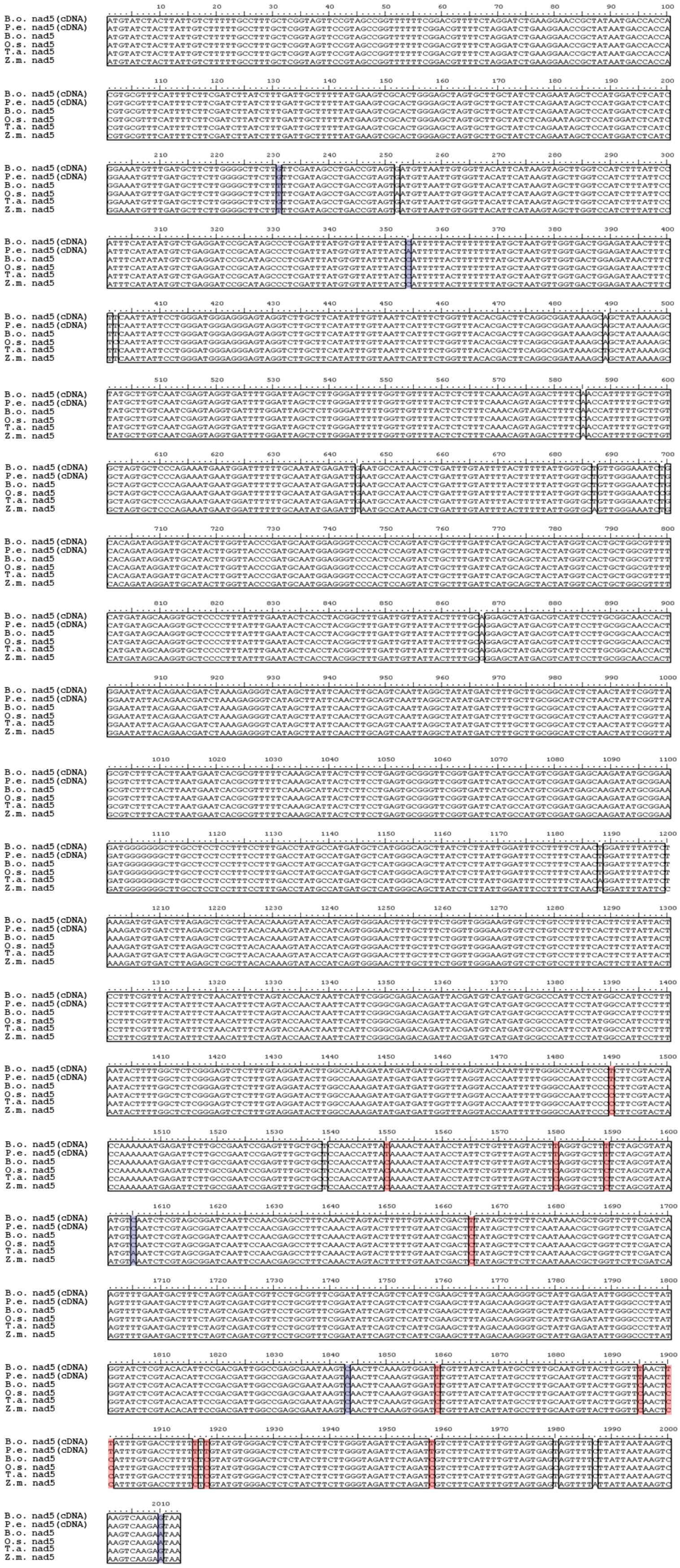

Figure S1 Comparison of bamboo mitochondrial *nad1*, *nad2*, *nad4* and *nad5* cDNA sequences with other species. The cDNA sequences of (A) *nad1*, (B) *nad2*, (C) *nad4*, and (D) *nad5* of *B. oldhamii* (B.o.) and *P. eduli* (P.e.) are compared with those of their homologues from monocotyledon *Oryza sativa* (O.S., DQ167399), *Triticum aestivum* (T.A., AP008982) and *Zea mays* (Z.M., AY506529) using the software BioEdit. The accession numbers of *nad1*, *nad2*, *nad4* and *nad5* were obtained from NCBI database. The sequences of mitochondrial genomic DNA (mtDNA) of *B. oldhamii* from Lin *et al.* [20] were also applied. The nucleotides which may proceed in C-to-U RNA editing were marked in red.

Figure S2

(A) NAD1.Bo (1)

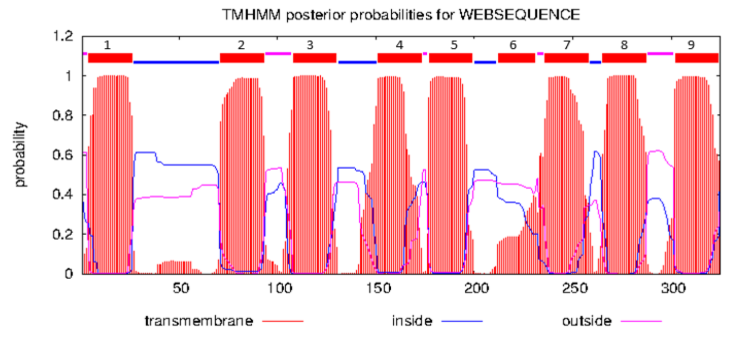

(B) NAD2.Bo (1)

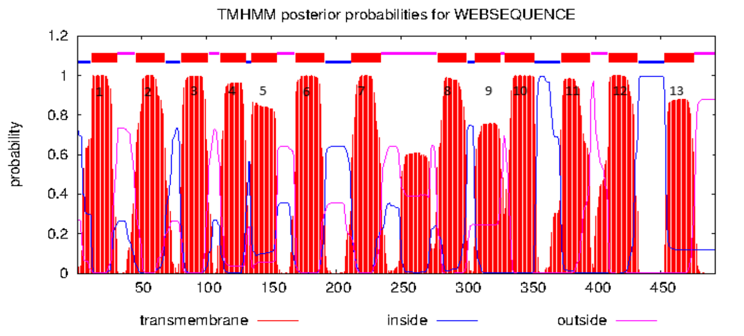

(C) NAD4.Bo (1)

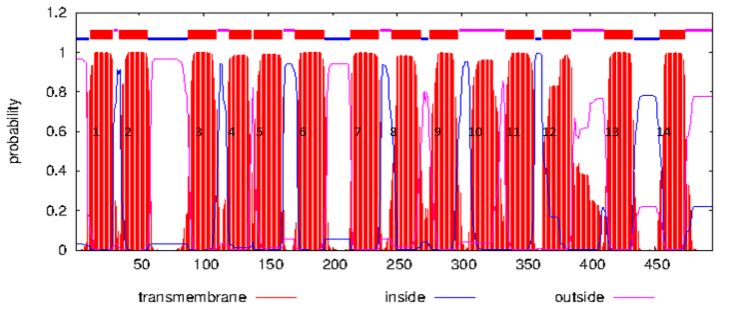

(D) Nad5-Bo (1)

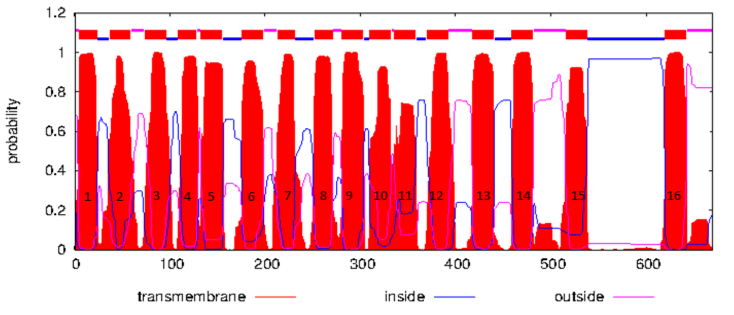

Figure S2 Prediction of the transmembrane helices of Nad1, Nad2, Nad4, and Nad5 subunits by TMHMM program. The transmembrane domain numbers of Nad1, Nad2, Nad4 and Nad5 subunits were based on the program TMHMM. (A) NAD1 subunit has 9 transmembrane helices, (B) NAD2 subunit has 13 (or 14) transmembrane helices, (C) NAD4 subunit has 14 transmembrane helices, and (D) NAD5 subunit has 16 transmembrane helices. Red line indicated transmembrane helices; blue and pink lines were inside and outside loops, respectively.

Figure S3A

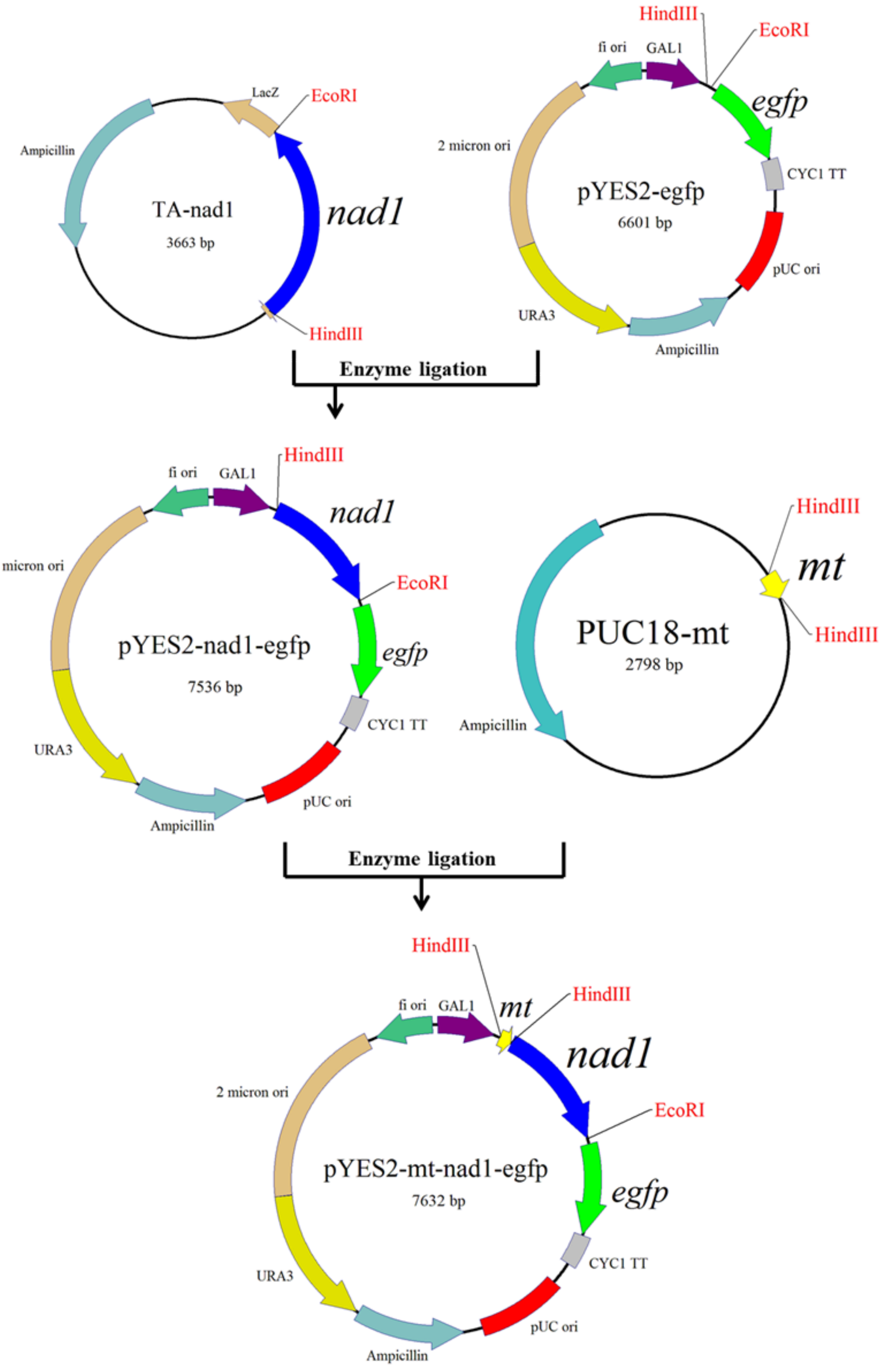

LacZ
EcoRI
Ampicillin
TA-nad1
3663 bp
nad1
HindIII
HindIII
EcoRI
fi ori
GAL1
egfp
2 micron ori
pYES2-egfp
6601 bp
CYC1 TT
pUC ori
URA3
Ampicillin
Enzyme ligation
HindIII
fi ori
GAL1
nad1
2 micron ori
EcoRI
pYES2-nad1-egfp
7536 bp
egfp
CYC1 TT
URA3
pUC ori
Ampicillin
HindIII
mt
PUC18-mt
2798 bp
HindIII
Ampicillin
Enzyme ligation
HindIII
HindIII
fi ori
GAL1
mt
nad1
2 micron ori
EcoRI
pYES2-mt-nad1-egfp
7632 bp
egfp
CYC1 TT
URA3
pUC ori
Ampicillin

Figure S3B

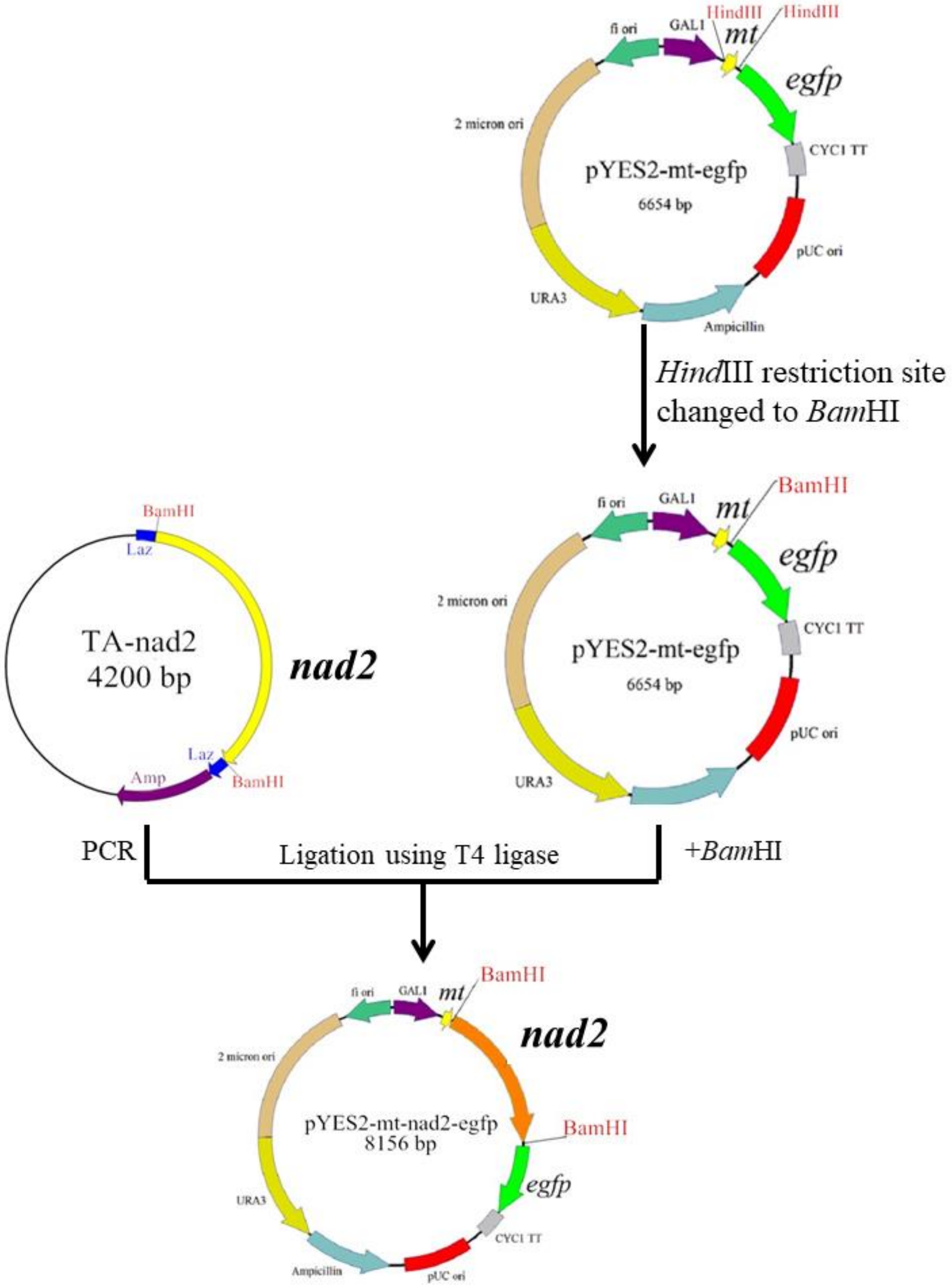
fi ori
GAL1
HindIII
HindIII
mt
egfp
2 micron ori
CYC1 TT
pYES2-mt-egfp
6654 bp
pUC ori
URA3
Ampicillin
HindIII restriction site
changed to BamHI
BamHI
Laz
TA-nad2
4200 bp
nad2
Laz
Amp
BamHI
fi ori
GAL1
mt
BamHI
egfp
2 micron ori
CYC1 TT
pYES2-mt-egfp
6654 bp
pUC ori
URA3
PCR
Ligation using T4 ligase
+BamHI
fi ori
GAL1
mt
BamHI
nad2
2 micron ori
pYES2-mt-nad2-egfp
8156 bp
BamHI
egfp
URA3
CYC1 TT
Ampicillin
pUC ori

Figure S3C

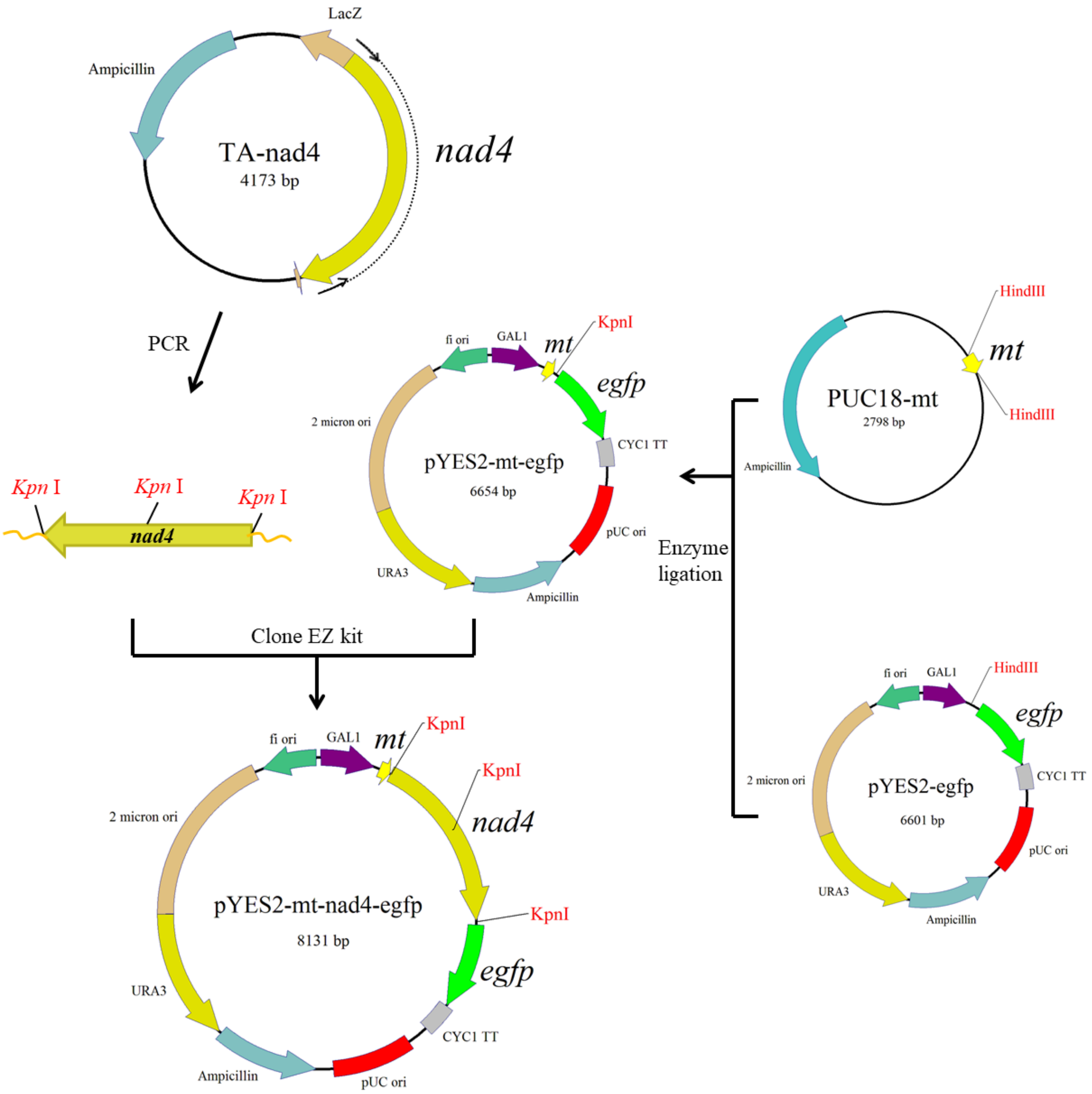

LacZ
Ampicillin
TA-nad4
4173 bp
nad4
PCR
Kpn I
Kpn I
Kpn I
nad4
fi ori
GAL1
mt
KpnI
egfp
2 micron ori
CYC1 TT
pYES2-mt-egfp
6654 bp
pUC ori
URA3
Ampicillin
HindIII
mt
PUC18-mt
2798 bp
HindIII
Ampicillin
Enzyme
ligation
Clone EZ kit
fi ori
GAL1
mt
KpnI
KpnI
nad4
2 micron ori
pYES2-mt-nad4-egfp
8131 bp
KpnI
egfp
URA3
CYC1 TT
Ampicillin
pUC ori
fi ori
GAL1
HindIII
egfp
2 micron ori
CYC1 TT
pYES2-egfp
6601 bp
pUC ori
URA3
Ampicillin

Figure S3D

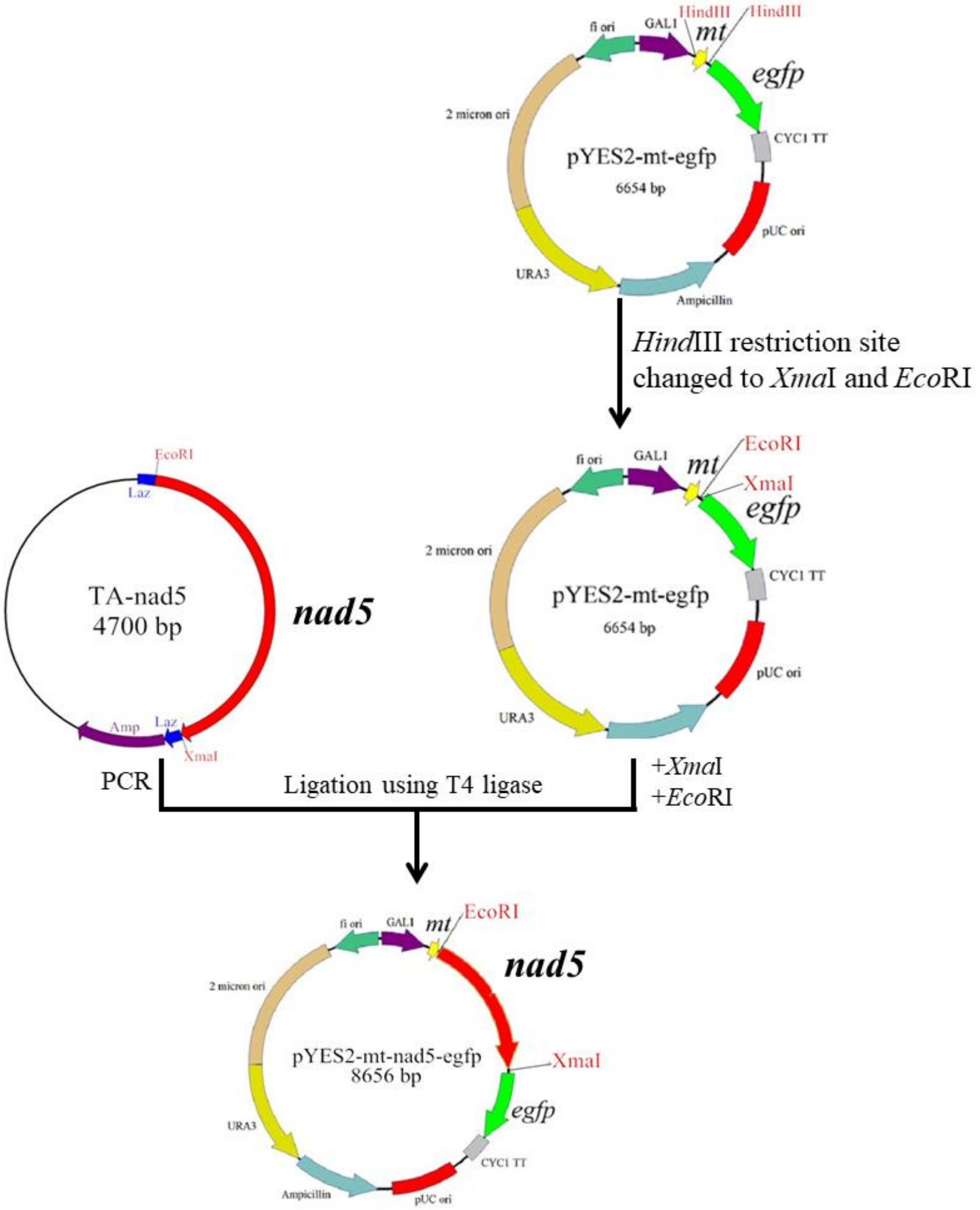

Figure S3 Schematic diagram of construction of pYES2-mt-nad1-egfp, pYES2-mt-nad2-egfp, pYES2-mt-nad4-egfp, and pYES2-mt-nad5-egfp plasmid. (A) The *nad1* fragment from TA-nad1 was ligated into pYES2-egfp to generate pYES2-nad1-egfp. The *mt* was ligated into pYES2-nad1-egfp

to generate pYES2-mt-nad1-egfp. (B) The *nad2* fragment from TA-nad2 was amplified by a pair of primers of BamHI-nad2-F and BamHI-nad2-R. The *nad2* fragment was ligated into pYES2-mt-egfp to generate pYES2-mt-nad2-egfp by using T4 ligase. (C) The *nad4* fragment from TA-nad4 was amplified by a pair of primers of nad4F-EZ and nad4R-EZ. The *nad4* fragment was ligated into pYES2-mt-egfp to generate pYES2-mt-nad4-egfp by using Clone EZ kit (GenScript). (D) The *nad5* fragment from TA-nad5 was amplified by a pair of primers of EcoRI-nad5-F and XmaI-nad5-R. The *nad5* fragment was ligated into pYES2-mt-egfp to generate pYES2-mt-nad5-egfp by using T4 ligase. *LacZ* : β-galactosidase gene; *mt* : mitochondrial targeting sequence; *egfp* : enhance green fluorescence protein; *GAL1* : yeast *GAL1* promoter; *CYC1 TT* : transcription terminator; pUC ori : E. coli replication origin bases; Ampicillin : ampicillin resistance gene; *URA3* : orotidine 5-phosphate decarboxylase; 2 micron ori : maintenance and high copy replication in yeast; fi ori : F1 phage origin.

Figure S4

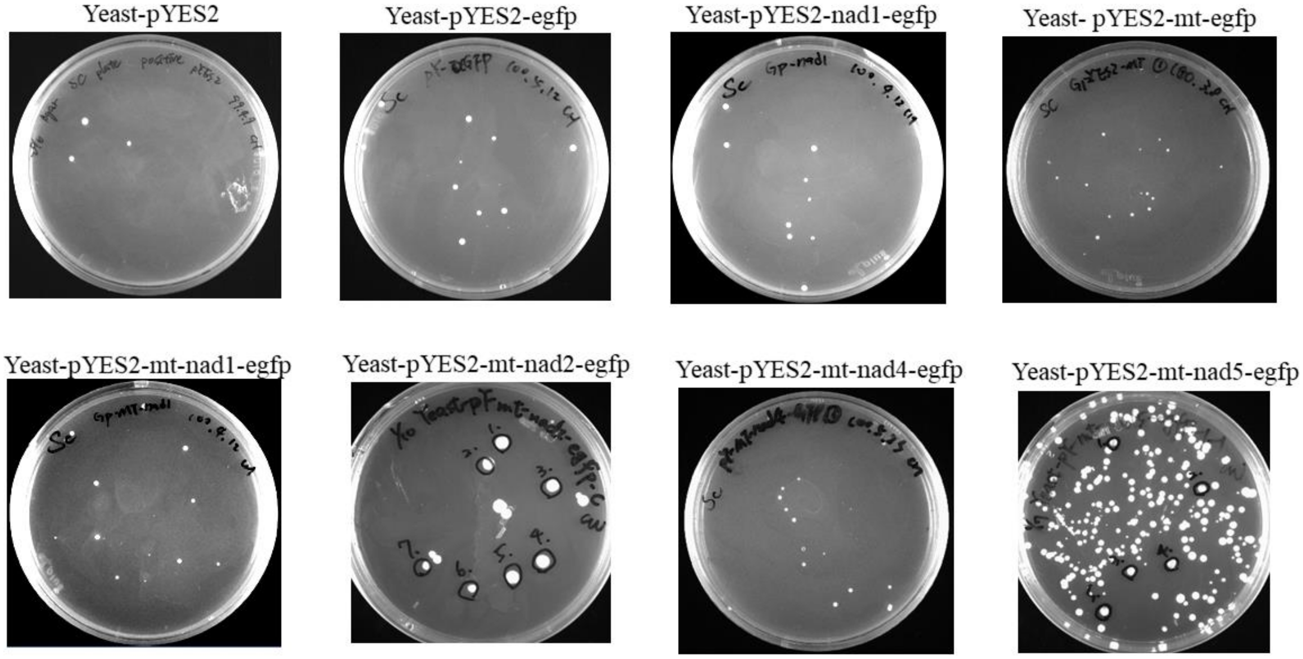

Figure S4 Selection of yeast transformants on SC-U medium plus 2% glucose plates. The transformants of Yeast-pYES2, Yeast-pYES22-egfp, Yeast-pYES2-nad1-egfp, Yeast-pYES2-mt-egfp, Yeast-pYES2-mt-nad1-egfp, Yeast-pYES2-mt-nad2-egfp, Yeast-pYES2-mt-nad4-egfp, pYES2-mt-nad5-egfp were spread on SC-U plate plus 2% glucose and cultured at 30°C. After 2 days, the presumptive transformants appeared as white colonies.

Figure S5

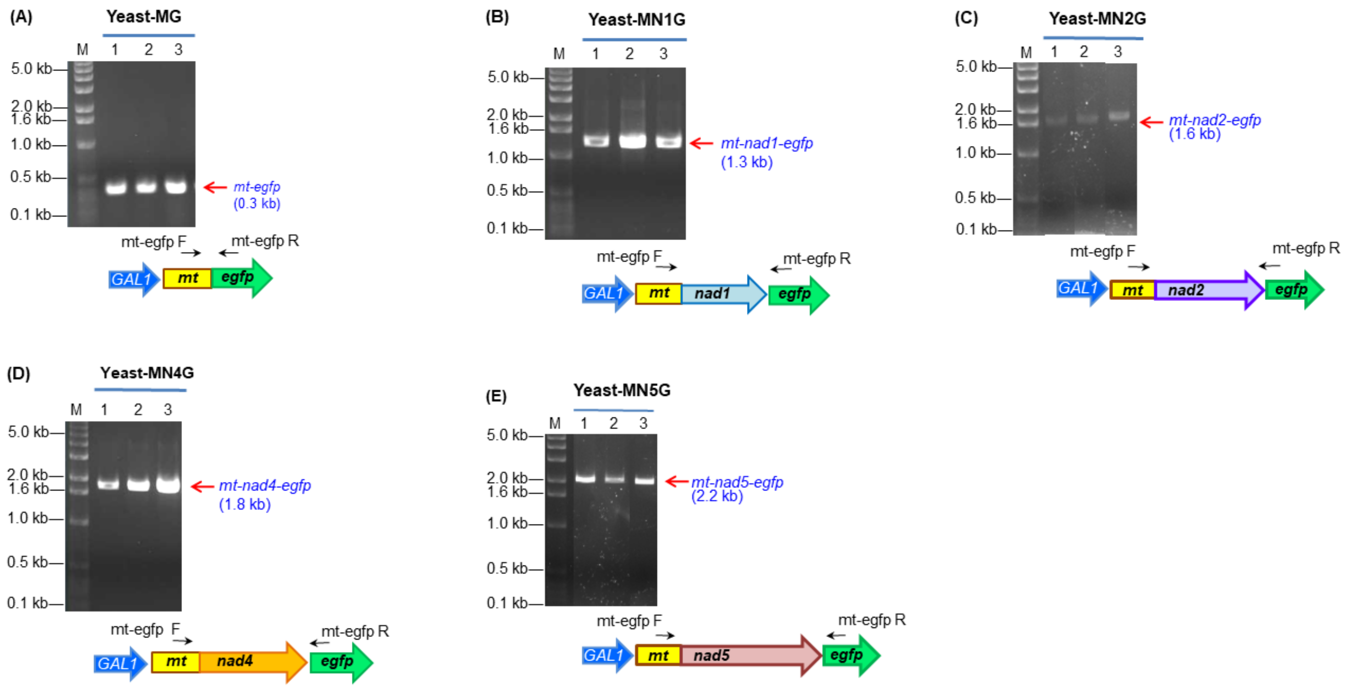

Figure S5 Detection of fusion genes in yeast transformants by colony PCR with a pair of primers mt-egfp-F and mt-egfp-R. (A) The 0.3 kb of *mt-efgp* fragments, (B) the 1.3 kb of *mt-nad1-egfp*, (C) the 1.6 kb of *mt-nad2-egfp*, (D) the 1.8 kb of *mt-nad4-dgfp*, and (E) the 2.2 kb of *mt-nad5-egfp* gene fragments were observed in the yeast transformants. M: 0.1 mg 1kb DNA ladders.

Figure S6

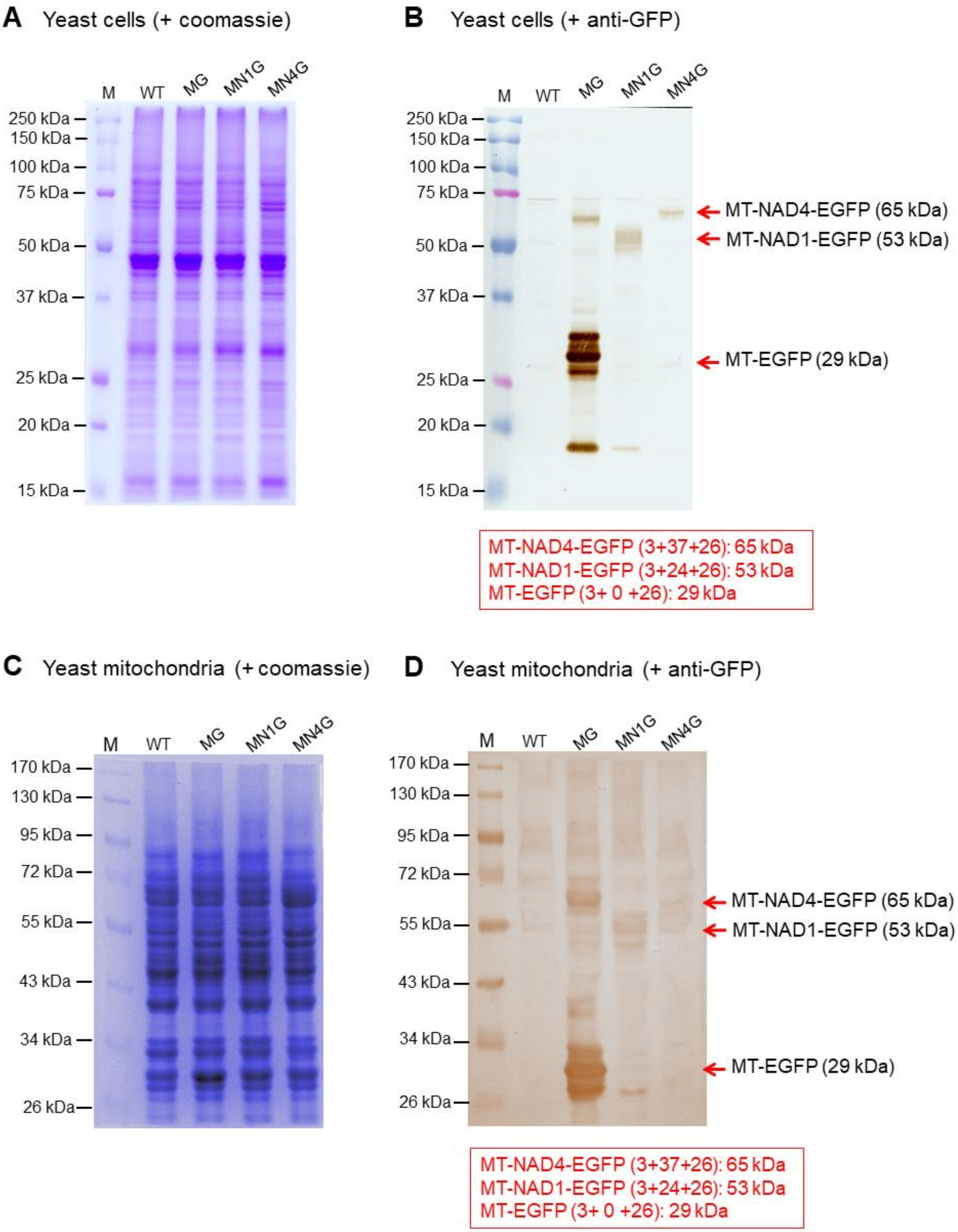
A Yeast cells (+ coomassie)
M WT MG MN1G MN4G
250 kDa
150 kDa
100 kDa
75 kDa
50 kDa
37 kDa
25 kDa
20 kDa
15 kDa
B Yeast cells (+ anti-GFP)
M WT MG MN1G MN4G
250 kDa
150 kDa
100 kDa
75 kDa
50 kDa
37 kDa
25 kDa
20 kDa
15 kDa
MT-NAD4-EGFP (65 kDa)
MT-NAD1-EGFP (53 kDa)
MT-EGFP (29 kDa)
MT-NAD4-EGFP (3+37+26): 65 kDa
MT-NAD1-EGFP (3+24+26): 53 kDa
MT-EGFP (3+ 0 +26): 29 kDa
C Yeast mitochondria (+ coomassie)
M WT MG MN1G MN4G
170 kDa
130 kDa
95 kDa
72 kDa
55 kDa
43 kDa
34 kDa
26 kDa
D Yeast mitochondria (+ anti-GFP)
M WT MG MN1G MN4G
170 kDa
130 kDa
95 kDa
72 kDa
55 kDa
43 kDa
34 kDa
26 kDa
MT-NAD4-EGFP (65 kDa)
MT-NAD1-EGFP (53 kDa)
MT-EGFP (29 kDa)
MT-NAD4-EGFP (3+37+26): 65 kDa
MT-NAD1-EGFP (3+24+26): 53 kDa
MT-EGFP (3+ 0 +26): 29 kDa

Figure S6 Detection of the expressed MT-EGFP, MT-NAD1-EGFP, and MT-NAD4-EGFP in yeast transformants with anti-GFP. (A) SDS-PAGE analysis of yeast total membrane proteins. Total membrane proteins of 25 μg from the transformants were separated on a 12% SDS-PAGE and stained with coomassie blue. (B) Extracted total membrane proteins were separated by SDS-PAGE, transferred to a PVDF membrane, and probed with anti-GFP (1:2000). The detected signals of MT-EGFP was 29 kDa, MT-NAD1-EGFP was 53 kDa, and MT-NAD4-EGFP was 65 kDa. (C) SDS-PAGE analysis of yeast mitochondrial membrane proteins. Mitochondrial membrane proteins of 25 μg from the transformants were separated on a 12% SDS-PAGE and stained with coomassie blue. (D) Extracted mitochondrial membrane proteins were separated by SDS-PAGE, transferred to a PVDF membrane, and probed with anti-GFP (1:2000). The detected signals of MT-EGFP was 29 kDa, MT-NAD1-EGFP was 53 kDa, and MT-NAD4-EGFP was 65 kDa. WT: wild type; MG: pYES2-mt-egfp transformant; MN1G: pYES2-mt-nad1-egfp transformant; MN4G: pYES2-mt-nad4-egfp transformant; M: prestained protein ladder (15-175 kDa or 26-170 kDa).

Figure S7

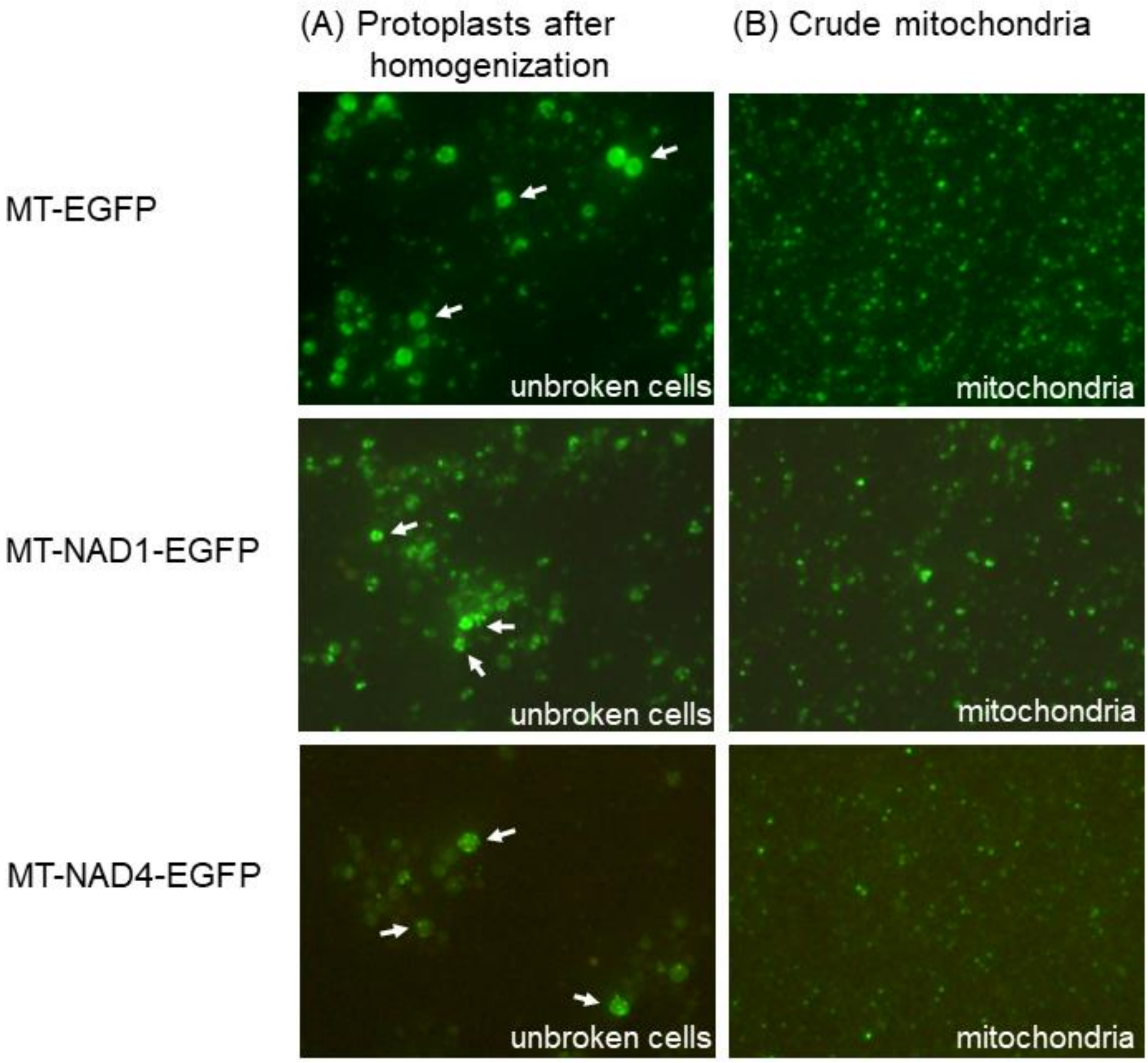

Figure S7 Observation of the yeast mitochondria containing EGFP under fluorescence microscopy. The yeast cells were broken by digestion with lyticase and by homogenization. They were observed under fluorescence microscope. (A) After homogenization, the unbroken yeast protoplasts (white arrows) and crude yeast mitochondria were recognized under fluorescence microscope (B) The crude yeast mitochondria were observed as green spots, after the cell debris and unbroken cells were removed.

Figure S8

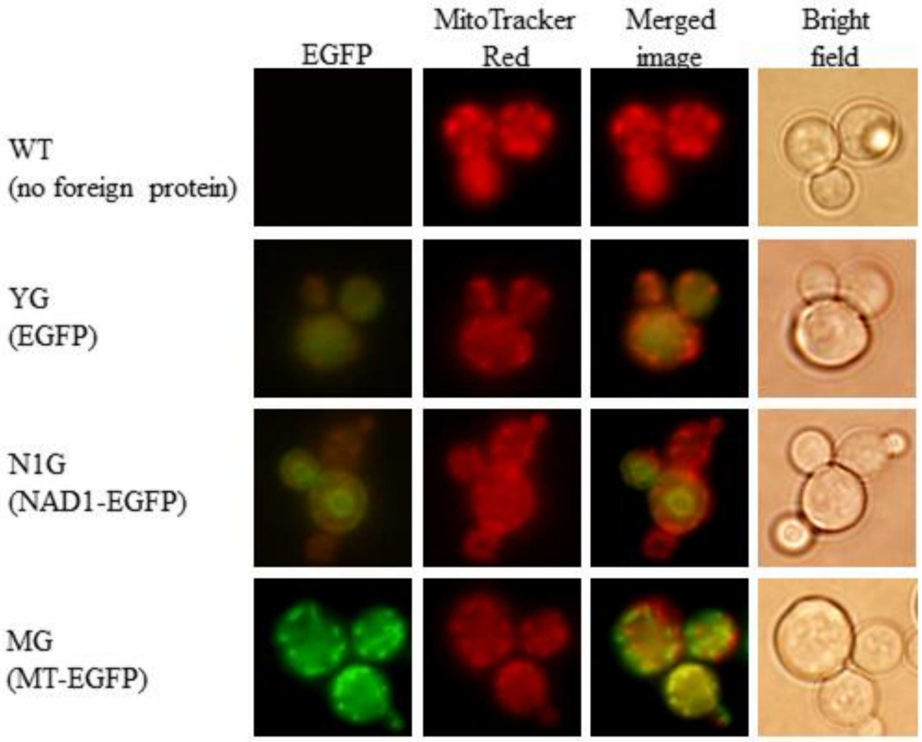

Figure S8. **Subcellular localization of EGFP, Nad1, and Nad1-EGFP fusion proteins after expression in yeast observed with fluorescence microscopy**. Yeast cells were incubated in SC-U medium plus 2% galactose for 15 h at 30°C to generate EGFP signal. Green fluorescence indicates the mitochondria containing EGFP, red fluorescence indicates the mitochondria labeled with ~~Mito Tracker~~ MitoTracker Red, and the yellow regions in the merged images indicate the co-occurrence in the mitochondria. The integrity of the cells was monitored by brightfield microscopy. WT: wild type; YG: yeast-pYES2-EGFP transformant; N1G: yeast-pYES2-Nad1-EGFP transformant; MG: yeast-pYES2-mt-egfp transformant.

Figure S9

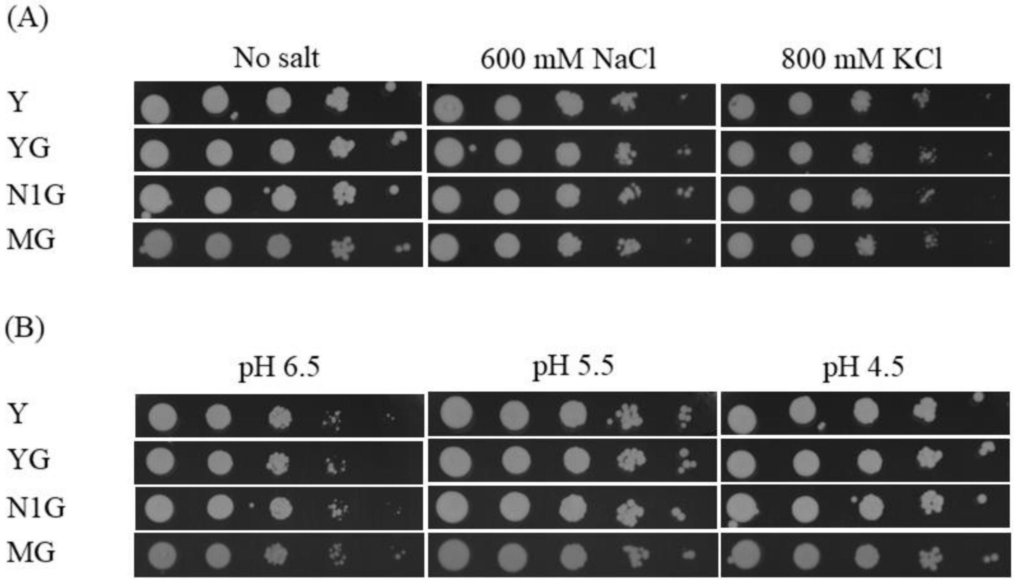

Figure S9. K^+, Na^+, or H^+ dependent growth phenotypes of yeast WT and transformants containing EGFP, NAD1-EGFP, and MT-EGFP. (A) Yeast cell cultures, grown to saturation for 24 h in SC-U medium plus 2 % glucose, were pipetted as a 1:10 dilution series onto SC-U plus 2 % galactose agar plates, with addition of no salt, 600 mM NaCl or 800 mM KCl. (B) Yeast cell cultures were treated as above but at pH 6.5, pH 5.5, or pH 4.5. Cell growth phenotypes were photographed after 3 days. Y: no foreign protein; YG: EGFP; N1G: NAD1-EGFP; MG: MT-EGFP.
